# Non-invasive quantification of collagen turnover in renal transplant recipients

**DOI:** 10.1371/journal.pone.0175898

**Published:** 2017-04-21

**Authors:** Elisabeth G. D. Stribos, Signe Holm Nielsen, Susanne Brix, Morten Asser Karsdal, Marc A. Seelen, Harry van Goor, Stephan J. L. Bakker, Peter Olinga, Henricus A. M. Mutsaers, Federica Genovese

**Affiliations:** 1 Department of Pharmaceutical Technology and Biopharmacy, University of Groningen, Groningen Research Institute of Pharmacy, Groningen, The Netherlands; 2 Department of Internal Medicine, Division of Nephrology, University of Groningen, University Medical Center Groningen, Groningen, The Netherlands; 3 Nordic Bioscience A/S, Herlev, Denmark; 4 Department of Biotechnology and Biomedicine, Technical University of Denmark, Lyngby, Denmark; 5 Department of Pathology and Medical Biology, Division of Pathology, University of Groningen, University Medical Center Groningen, Groningen, The Netherlands; University of Campinas, BRAZIL

## Abstract

Kidney allograft failure due to chronic injury/rejection remains the main cause of graft loss in renal transplant recipients (RTR). Here, we investigated whether specific biomarkers of extracellular matrix (ECM) turnover are associated with allograft function and chronic kidney disease (CKD) stage in RTR. Seventy-eight patients who attended the University Medical Center Groningen for a routine check-up after kidney transplantation were enrolled in the study. Plasma and/or 24h-urine samples were collected and specific matrix-metalloproteinase-generated neo-epitope fragments of collagens were measured by enzyme-linked immunosorbent assay. Our results demonstrated that urinary levels of C3M, a marker for collagen type III degradation, correlated with estimated glomerular filtration rate (eGFR; r = 0.58, p<0.0001), with lower levels detected in the urine of patients with advanced CKD. In addition, plasma levels of Pro-C6, a marker for collagen type VI formation, significantly increased with disease progression and correlated with eGFR (r = -0.72, p<0.0001). Conversely, plasma C3M and urinary Pro-C6 levels showed no correlation with renal function. We identified two neo-epitope biomarkers of tissue turnover associated with ECM remodeling and fibrosis that can stratify patients by CKD stage. This is as promising first step towards non-invasive monitoring of ECM turnover in the kidneys.

## Introduction

Progressive loss of kidney allograft function due to fibrosis greatly hampers long-term allograft survival and remains a major hurdle in renal transplantation. Allograft loss is closely related to interstitial fibrosis and tubular atrophy (IF/TA), and histological features of this pathological process can already be detected at 3–6 months after transplantation in 40% of kidney allografts [1–3]. Moreover, the presence of these morphological features is associated with poorer allograft survival [4]. Renal allograft fibrosis reflects a pathological response to (tubular) injury where the equilibrium between extracellular matrix (ECM) formation and degradation is deregulated [5,6].

Currently, biopsies are the gold standard for the diagnosis of renal fibrosis, allowing early detection of pathological changes before clinical features, such as diminished renal function and proteinuria, become apparent. Nevertheless, protocol biopsies are invasive, with discomfort for the patients and prone to sampling variability and errors [7]. Due to the abundance of collagens in connective tissues and organs, they are interesting biomarker candidates. Consequently, several studies have focused on the detection of urine- and blood-based biomarkers for diagnosis and prognosis of kidney diseases during the last decade [8]. Protein fingerprint biomarkers of collagens and other ECM proteins, reflecting ECM remodeling, have already been proven to be useful as both prognostic and diagnostic biomarkers in patients with hepatic fibrosis, idiopathic pulmonary fibrosis and undergoing hemodialysis [6,9,10]. However, the identification of ECM turnover markers as a biomarker of fibrosis in the transplanted kidney has not been widely studied. It has been demonstrated that serum and urine levels of the collagen type III pro-peptide (surrogate markers for active collagen formation) are increased during renal fibrosis [11–13]. In addition, urinary collagen type IV has been associated with loss of renal function [14–17]. Such non-invasive biomarkers could enable frequent monitoring of renal patients and facilitate clinical trials studying the effect of antifibrotic therapies.

In the present study, we evaluated different biomarkers of collagen degradation and formation to define a protein fingerprint associated renal allograft function and CKD stage in renal transplant recipients (RTR).

## Materials and methods

### Data collection

Lithium-heparin plasma and/or 24h-urine was collected at the outpatient clinic of the University Medical Center Groningen (UMCG) from 78 RTR during a routine check-up. Plasma samples were collected from 75 RTR and 24h-urine samples were collected from 42 RTR; these numbers include 39-paired plasma and urine samples. Samples were stored at -80°C until analysis. Creatinine, protein, sodium (both in plasma and urine) and creatinine clearance were measured by the UMCG clinical laboratory as standard procedure. Estimated glomerular filtration rate (eGFR) was calculated based on the Modification of Diet in Renal Disease (MDRD) formula. This study was approved by the Medical Ethical Committee of the UMCG, according to Dutch legislation and the Code of Conduct for dealing responsibly with human tissue material in the context of health research (www.federa.org), refraining the need of written consent for ‘further use’ of coded-anonymous human material. The procedures were carried out in accordance with the experimental protocols approved by the Medical Ethical Committee of the UMCG (METC number 2014/077).

### Biomarker measurements by enzyme-linked immunosorbent assay (ELISA)

The panel of biomarkers included both formation and degradation markers of collagens measured by competitive ELISAs developed by Nordic Bioscience (Herlev, Denmark) as specified in Table 1. The ELISAs were performed as previously described (see references in Table 1 for the single assays). Briefly, a 96-well ELISA plate purchased coated with streptavidin cat. 11940279 (Roche, Hvidovre, Denmark), was coated with the synthetic peptide at 20°C for 30 min by constant shaking at 300 rpm. The plate was then washed five times in washing buffer (20mM Tris, 50 mM NaCl, pH 7.2). Thereafter, 20 μl of the standard peptide or sample diluted according to protocol was added, followed by 100 μl of peroxidase conjugated anti-human mAb. The plate was incubated 1 h at 20°C or overnight at 4°C while shaking at 300 rpm (according to protocol). Afterwards, the plate was washed five times. Finally, 100 μl TMB (Kem-En-Tec, Taastrup, Denmark) was added and the plate was incubated for 15 min in the dark while shaking at 300 rpm. To end the reaction, 100 μl of stopping solution (1% H_2_SO_4_) was added and the plate was analyzed on the ELISA reader at 450 nm with 650 nm as the reference. All samples were measured within the detection range of the assay. Samples below lower limit of quantification (LLOQ) were assigned the value of LLOQ. The urinary marker levels were normalized for urine creatinine concentration, measured with QuantiChrom Creatinine Assay Kit (Bioassay Systems, Hayward, USA).

**Table 1:** Overview of measured biomarkers to assess ECM turnover in plasma and/or urine.

Biomarker	Target	Detection range (ng/mL)	Intra- and inter-assay variation (%)	Urine/plasma	Reference
**C1M**	MMP-mediated degradation of collagen type I (alpha 1 chain)	11.82–133.0	5.0 and 7.0	Urine	[18]
**C3M**	MMP-mediated degradation of collagen type III	2.0–59	9.5 and 3.5	Urine and Plasma	[19,20]
**Pro-C3**	Formation of collagen type III	2.66–48.20	6.5 and 12.4	Urine and Plasma	[21]
**C4M**	MMP-mediated of collagen type IV (alpha 1 chain)	5.7–187	5.3 and 2.8	Urine and Plasma	[22]
**C5M**	MMP-mediated degradation of collagen type V	1.29–14	9.4 and 5.8	Plasma	[23]
**Pro-C6**	Formation of collagen type VI	0.602–97.23	4.0 and 12.7	Urine and Plasma	[24]
**C6M**	MMP-mediated degradation of collagen type VI	3–210	10.0 and 7.0	Plasma	[25]

### Total collagen type VI and Picro-Sirius Red (PSR) staining

Expression of collagen type VI in human renal tissue was studied by immunohistochemistry. Healthy renal tissue (n = 3) was obtained from kidneys removed due to renal cell carcinoma (RCC). In addition, fibrotic renal tissue was obtained from anonymous chronic rejection biopsy samples since tissue samples were not available from the RTR cohort. Tissue sections (2 or 4 μm) were deparaffinized and rehydrated in successive baths of xylene and graded ethanols (99%-70%). Afterwards, the slides were heated in sodium citrate buffer (pH 6) at 100°C for 15 min. Endogenous peroxidase was blocked with 0.09% H_2_O_2_ for 30 min. Next, non-specific epitopes were blocked using 10% normal donkey serum and subsequently incubated with rabbit-anti-collagen VI antibody (1:150 or 1:100, ab6588, Abcam, Cambridge, UK). Goat anti-rabbit and HRP-conjugated rabbit anti-goat antibodies in 1% normal human serum were used as secondary and tertiary antibodies, respectively (both 1:100, Dako, Glostrup, Denmark). All antibodies were diluted in PBS containing 1% BSA. Detection was performed with aminoethyl carbazole (AEC), counterstained with Mayer’s hematoxylin (Merck, Darmstadt, Germany) and analyzed by light microscopy (Leica DM2000 LED). The Picro-Sirius Red (PSR) stainings were performed on paraffin sections.

### Statistics

Differences between the biomarker levels stratified into CKD stages, were determined using non-parametric Kruskal-Wallis test and Dunn’s multiple comparison post-hoc test. Statistics on parametric data (plasma Pro-C6 and patient demographics) were performed using one-way ANOVA and the Chi-square test was used for categorical variables. Mean values, standard error of the mean (SEM) and the area under the curve (AUC) were calculated using MedCalc (MedCalc, Belgium). Correlations between biomarkers and eGFR were calculated using GraphPad Prism, version 6 (GraphPad Software, San Diego, CA, USA). Statistically significance threshold was set at p<0.05 and data were presented as mean ± SEM where appropriate.

## Results

### Cohort characteristics

A total of 78 renal transplant patients (48 men and 30 women) with a median age of 52 years (20 to 83 years) and a median time after transplantation of 5.41 years (0.05 to 39.36 years) were enrolled in this study. Primary renal disease of these patients were chronic renal failure without known cause (n = 13, 16.7%), cystic renal disease (n = 12, 15.4%), hypertension or renal vascular disease (n = 10, 12.8%), pyelonephritis/interstitial nephritis (n = 10, 12.8%), diabetic nephropathy (n = 9, 11.5%), IgA glomerulopathy (n = 8, 10.3%) and other diagnosis or missing data (n = 16, 20.5%). The patients were categorized into chronic kidney disease (CKD) stages 2–4 by Modification of Diet in Renal Disease (MDRD) for analysis as shown in Table 2. The only patient with CKD stage 1 was excluded from categorical analysis because of low statistical power.

**Table 2:** Patient characteristics of the study population stratified by CKD stages (n = 77).

Variable	CKD stage 2 (n = 19)	CKD stage 3A (n = 23)	CKD stage 3B (n = 19)	CKD stage 4 (n = 16)	P-value^a^
**Age (years), mean ± SD**	54.15 ± 17.03	51.21 ± 13.04	51.75 ± 12.42	52.09 ± 13.56	0.88
**Male, n (%)**	11 (57.9%)	14 (60.9%)	11 (57.9%)	11 (68.8%)	0.91
**eGFR, mean ± SD**	69.47 ± 7.86	51.21 ± 4.17	36.74 ± 3.77	23.56 ± 4.22	<0.001
**Time after transplant, years ± SD**	6.99 ± 7.86	1.78 ± 3.06	8.36 ± 12.49	5.77 ± 5.62	0.02
**Urinary protein g/24h**	0.17 ± 0.11	0.25 ± 0.34	0.27 ± 0.21	1.25 ± 1.39	<0.001
**Urinary sodium mmol/24h**	172.4 ± 63.6	169.8 ± 67.8	161.2 ± 79.1	86.1 ± 34.6	0.002
**Serum Creatinine (μmol/L)**	90.0 ± 14.9	118.1 ± 14.5	156.8 ± 24.2	241.6 ± 44.0	<0.001
**Urine Creatinine mmol/24h**	11.8 ± 3.9	11.6 ± 3.3	10.6 ± 3.4	10.1 ± 2.0	0.43
**Urea mmol/24h**	396.0 ± 129.6	372.4 ± 132.5	368.1 ± 184.6	257.9 ± 54.1	0.05
**Creatinine clearance (mL/min)**	91.1 ± 24.4	68.3 ± 16.6	47.5± 12.4	29.8 ± 9.1	<0.001
**Immunosuppresive regimen:**					0.71
*I—Standard triple therapy*	12 (63%)	16 (73%)	12 (63%)	10 (63%)	
*II—Standard dual therapy*	1 (5%)	1 (4.5%)	1 (5%)	0 (0%)	
*III—Dual therapy with CNI*	3 (16%)	1 (4.5%)	3 (16%)	4 (25%)	
*IV—Dual therapy with azathioprine*	1 (5%)	0 (0%)	2 (11%)	0 (0%)	
*V—Triple therapy*, *other*	2 (11%)	4 (18%)	1 (5%)	2 (12%)	
**Immunosuppressive regimen with calcineurin inhibitor (CNI)**	16 (84%)	22 (100%)	16 (84%)	16 (100%)	0.09
**Antihypertensive drugs**	16 (84%)	15 (68%)	18 (95%)	13 (81%)	0.18
**Antiglycaemic drugs**	4 (21%)	6 (21%)	4 (31%)	5 (27%)	0.87
**Lipid lowering drugs**	6 (32%)	9 (41%)	8 (42%)	6 (38%)	0.91
**Acute rejection rate**	3 (16%)	1 (5%)	2 (11%)	1 (6%)	0.62

^**a**^ Comparisons between patient characteristics were performed using one-way ANOVA with Student-Newman-Keuls Post-hoc test and Chi-square test for categorical variables.

### Urinary marker of collagen type III degradation associated with eGFR

**Fig 1:**
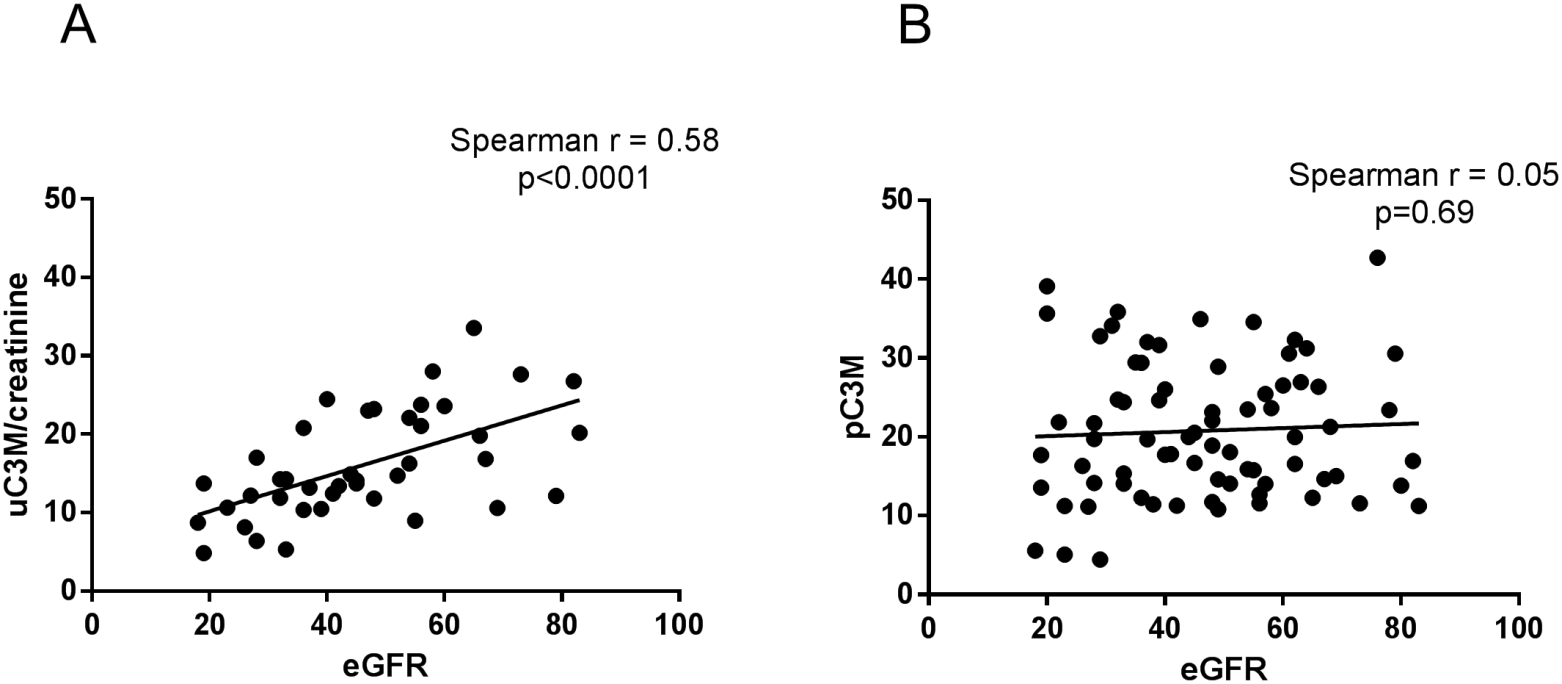
Associations between estimated glomerular filtration rate (eGFR) and biomarkers for ECM remodeling in urine and plasma. Correlations of creatinine-normalized urinary C3M (uC3M/creatinine) (A) and plasma C3M (pC3M) (B) with eGFR in RTR.

Among the measured urinary markers, uC3M/creatinine, reflecting degradation of collagen type III, significantly correlated with eGFR (Spearman r = 0.58, p<0.0001; Fig 1A). Specifically, the uC3M/creatinine levels were lower in urine of patients with poor kidney function (S1 Fig). The same marker measured in plasma (pC3M) could not stratify RTR per CKD stage, and was not correlated with eGFR (Spearman r = 0.05, p = 0.69; Fig 1B and S1 Fig). The calculated diagnostic value of uC3M/creatinine for separation of mild and moderate CKD (stage 2, 3A and 3B) from severe CKD (stage 4) resulted in an AUC of 0.83 (p = 0.004). Levels of uC3M did not correlate with recipient age, gender or transplant era (S1 Table).

### Plasma marker of collagen type VI formation is associated with eGFR

**Fig 2:**
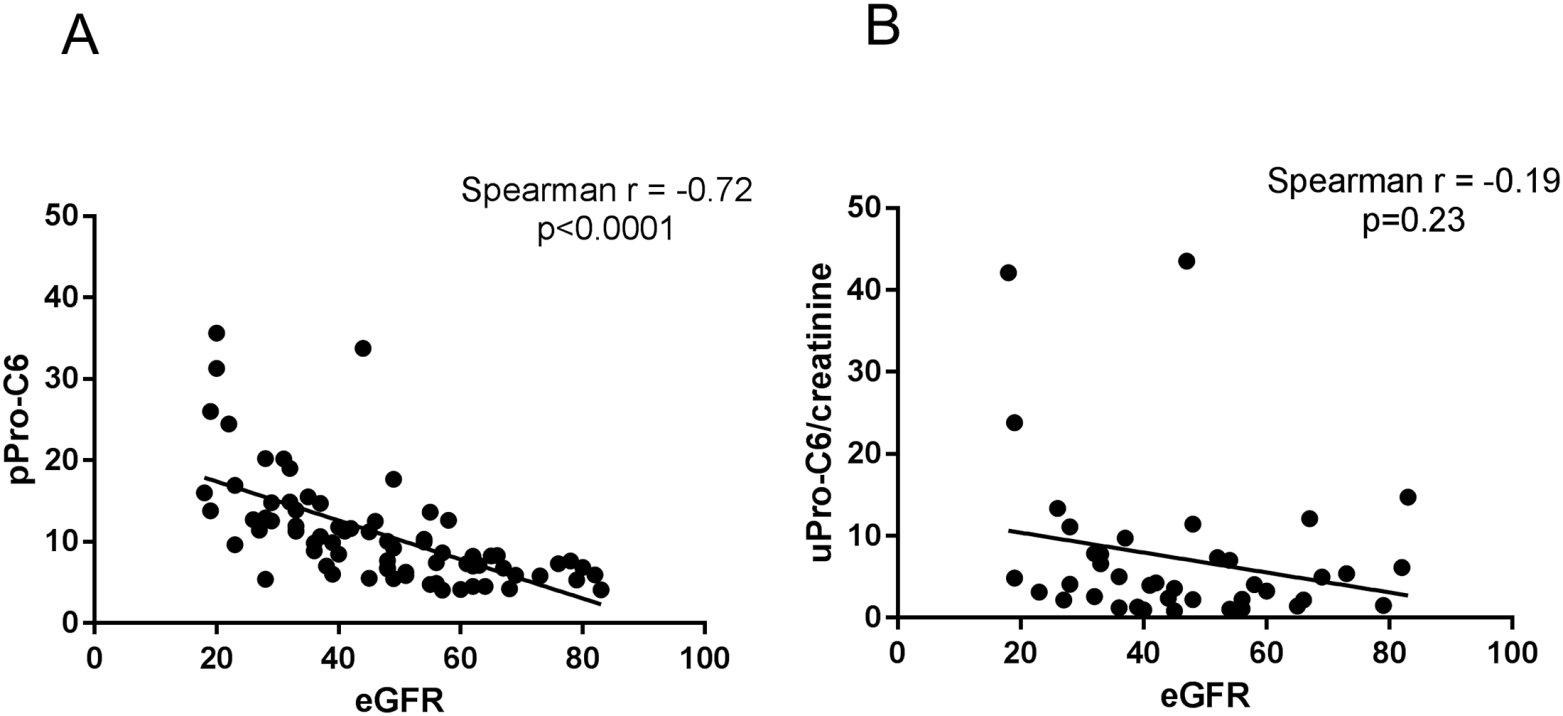
Associations between estimated glomerular filtration rate (eGFR) and biomarkers for ECM remodeling in urine and plasma. Correlations of urinary uPro-C6 normalized to creatinine (uPro-C6/creatinine) (A) and plasma Pro-C6 (pPro-C6) (B) with eGFR in RTR.

Among the measured plasma markers, pPro-C6, reflecting formation of collagen type VI, strongly correlated with eGFR (Spearman r = -0.72, p<0.0001; Fig 2A). This was not found for urinary Pro-C6 normalized for creatinine (uPro-C6/creatinine, Spearman r = -0.19, p = 0.23; Fig 2B). In addition, pPro-C6 levels reflected CKD stage, with levels increasing with disease severity (S1 Fig); this association was again not observed for uPro-C6/creatinine (S1 Fig). The prognostic value for separation of mild to moderate CKD (stage 2, 3A and 3B) from severe CKD (stage 4) resulted in an AUC of 0.84 (p<0.0001). Levels of pPro-C6 did not correlated with recipient age, gender or transplant era (S1 Table). Of note, the other measured biomarkers (C1M, C3M, Pro-C3, C4M, C5M, Pro-C6 and C6M) did not correlate with renal function (S1 Table, S1 Dataset).

### Expression of collagen type VI was increased in fibrotic kidney tissue

**Fig 3:**
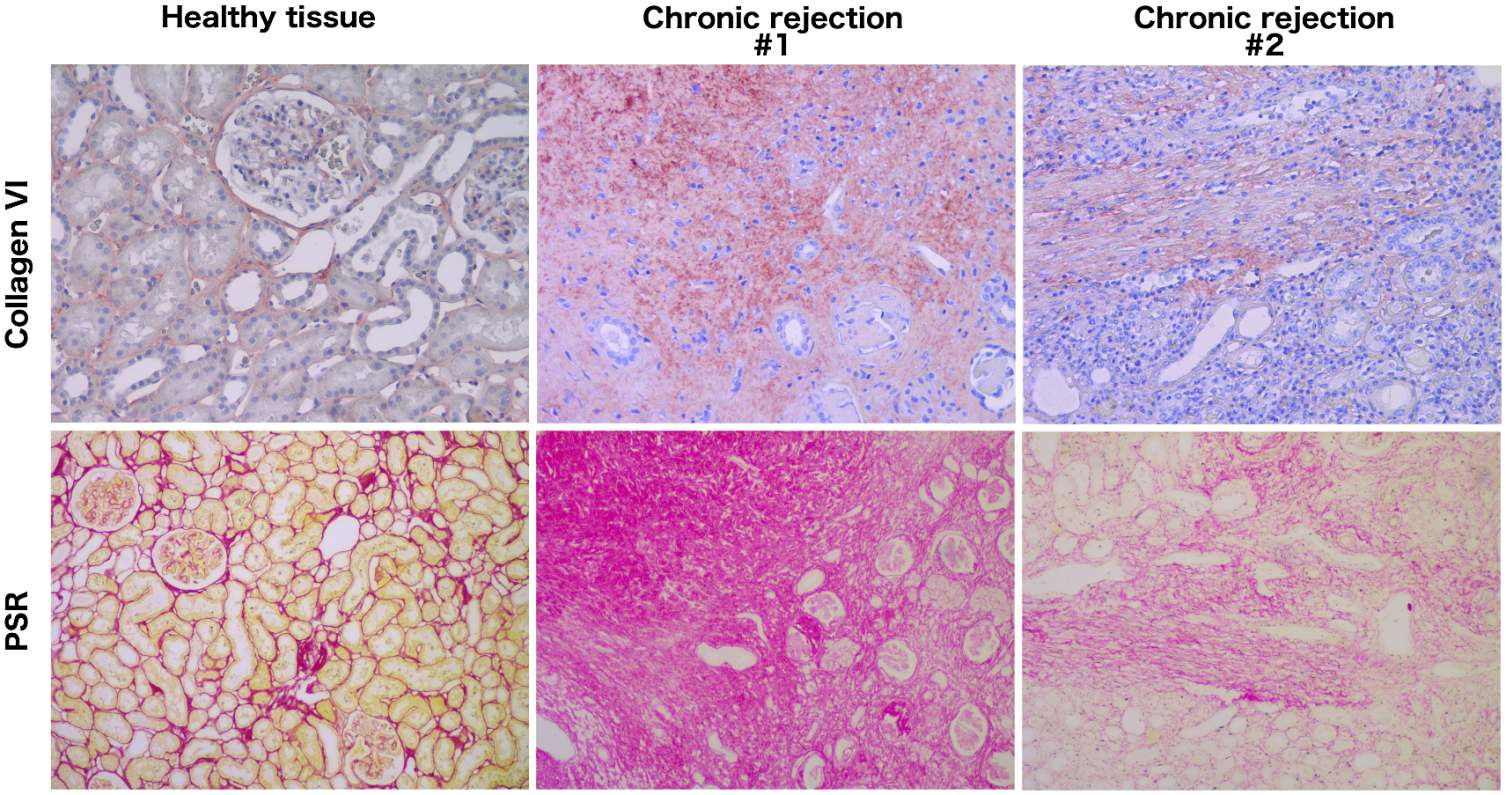
Representative images of collagen type VI staining in healthy and fibrotic renal tissue. Magnification 200x for collagen VI staining and 100x for PSR staining. PSR, Picro-Sirius Red.

As renal expression of collagen type VI has been poorly investigated, we studied its localization in healthy as well as fibrotic renal tissue (Fig 3). In healthy tissue (obtained from the microscopically healthy part of an RCC kidney), collagen type VI was localized mainly in the interstitium, and to a lesser extent in the Bowman capsule and the renal vasculature (primarily the adventitial layer). Moreover, an intense collagen type VI staining was observed in fibrous lesions in chronic rejection biopsies. The extent of fibrosis was confirmed by PSR staining which is in line with the observed localization of collagen VI.

## Discussion

Non-invasive methods to provide information on disease stage or prognosis of renal loss in transplanted kidneys are currently unavailable [26]. Markers of ECM protein turnover are good candidates as diagnostic and prognostic biomarkers, since renal allograft loss is closely associated with, and possibly a consequence of, renal fibrosis. Moreover, ECM markers have shown a great prognostic potential in fibrotic disease in other organs [10,27]. In the present study, performed in a cohort of RTR, we demonstrated that (1) low levels of the collagen type III degradation marker C3M in urine is associated with a poor kidney function and (2) high levels of the collagen type VI formation marker Pro-C6 in plasma is associated with reduced kidney function.

The observation that uC3M progressively decreases in advancing CKD stages, while plasma levels remain stable, is in agreement with previous observations in IgA nephropathy patients [20]. Thus, C3M measured in a blood sample does not have the same diagnostic value as when measured in urine. This may be because circulating C3M does not reflect glomerular and tubular damage occurring in advanced stages of CKD, but rather a systemic inflammatory state, as postulated previously [20]. Unfortunately, the excretion route (*i*.*e*. glomerular filtration or tubular excretion) of the measured biomarkers is unknown. Thus, it is unclear if and how glomerular/tubular damage and residual function of the native kidney influence uC3M levels. Nonetheless, the use of uC3M as a diagnostic marker for renal disease is supported by a urinary proteomics study, in which fragments of collagen type III, including those detected by the uC3M assay, were shown to be potential markers for IF/TA in CKD patients [26]. The same authors also showed that measurements of collagen type III fragments could serve as potential diagnostic markers for acute rejection and graft injury [26]. This, as well as the current findings, supports the relevance of urinary collagen fragments as non-invasive biomarkers for the diagnosis of renal fibrosis following renal transplantation.

Collagen type VI is mainly found in interstitial connective tissues as a structural component of microfibrils [20,28,29], and it is well known that this protein is instrumental for muscle function [30]. The presence of collagen type VI in the kidney was already demonstrated in the 1990s by immunohistochemical analysis [31,32], and more recent mass spectrometry-based proteomic studies showed that it is one of the most abundant proteins in the glomerular ECM [33]. The observed distribution of collagen type VI within the healthy kidney in the present study is in line with the classic studies from the 1990’s showing minimal staining in the interstitium, the glomerulus and renal vasculature. Almost no studies exist on the expression pattern of collagen type VI in renal pathologies. The ascertained increase of collagen type VI in the fibrous lesions as well as the chronic rejection biopsies corroborates previous findings in a range of patients with glomerular disorders and inflammatory renal diseases [31,34]. Taken together, the increase of collagen type VI in fibrotic lesions as well as the progressive increase of Pro-C6 in the plasma of RTR suggest that this protein can be a useful marker to study renal ECM turnover.

Our results show differences in ECM turnover in RTR patients correlating with renal function, which is in agreement with MMP and TIMP imbalance described in renal allografts. Racca *et al*. showed higher levels of proMMP9, the mediator of collagen III degradation, in RTR with IF/TA and levels were inversely correlated with eGFR [35]. Others also studied MMP and TIMP expression during renal transplantation in urine, plasma and biopsies. However, due to the minimal amount of publications on this specific topic, no hard conclusions can be drawn yet although it seems that MMP9, TIMP1 and 2 increase with fibrogenesis [36–39].

In this study we identified two novel biomarkers that can possibly be used to non-invasively describe the fibrotic process in the kidney; these markers could potentially decrease the need for kidney biopsies in the future. Moreover, being dynamic markers of tissue remodeling, these biomarkers could have prognostic value, identifying patients with more active disease. These findings are a promising first step towards non-invasive monitoring of extracellular matrix turnover in renal allografts, which will in the long run aid in the search for novel antifibrotic therapies. Nonetheless, the current study has some limitations. First of all, the relatively small cohort used in this study was very diverse, thus limiting the value of calculating CKD stage reference values for urinary and plasma levels of C3M and Pro-C6, respectively. Future studies would benefit from inclusion of patients that are less varied in factors such as time since transplantation, and inclusion of longitudinal follow-up, to evaluate the ECM remodeling markers in urine and circulation over time. Ideally, it would be of additional value to have an IF/TA score based on observation of kidney biopsies taken at time of sampling. Another limitation involves the marker specificity related to the ECM products. Since collagen type III is expressed in soft tissues throughout the body [40], C3M cannot be qualified as organ specific. The same is seen with collagen type VI, which is also found in other organs from which it may be released into circulation [41,42]. In addition, the observational study design limits the ability to infer causation.

In conclusion, our study shows that urinary C3M and plasma Pro-C6 are promising markers of collagen turnover in RTR. Further studies are needed to ascertain their clinical role in disease progression as well as in the search for novel antifibrotic therapies.

## Supporting information

S1 Table Correlations of all measured biomarkers with each other, age, gender, eGFR, urinary protein and transplant era (expressed as Spearman rank correlation coefficient and p values). (DOCX)

S1 Fig CKD stages stratification for urinary and plasma C3M and Pro-C6. Levels of uC3M/creatinine (A), pC3M (B), uPro-C6/creatinine (C) and pPro-C6 (D) in RTR divided into CKD stage 2 to 4. Statistical differences were assessed by Kruskal Wallis test and Dunn's multiple comparison post-hoc test. Asterisks indicate statistical significance between specified groups as indicated by bars. (* = p<0.05; ** = p<0.01; **** = P<0.0001).
(TIF)

S1 Dataset (XLSX)
