# Supplementary material for: Non-invasive quantification of collagen turnover in renal transplant recipients
Source: PLoS One. 2017 Apr 21;12(4):e0175898. doi: 10.1371/journal.pone.0175898 (PMC5400243; doi:10.1371/journal.pone.0175898)
Supplement: S1 Table — (DOCX) [file pone.0175898.s001.docx]

**Table 3.** Correlations of all measured biomarkers with each other, age, gender, eGFR, urinary protein and transplant era (expressed as Spearman rank correlation coefficient and p values).

|  | | Age | Gender | eGFR | Urinary protein | Transplant era | C3M | C4M2 | C5M | C6M | Pro-C6 | uC3M | uPro-C6 |
| --- | --- | --- | --- | --- | --- | --- | --- | --- | --- | --- | --- | --- | --- |
| Age | Correlation Coefficient Significance Level P n |  | -0.232 0.0425 77 | 0.183 0.1114 77 | -0.145 0.2466 66 | -0.129 0.2672 76 | -0.128 0.2776 74 | -0.101 0.3902 74 | 0.106 0.3684 74 | -0.140 0.2355 74 | -0.125 0.2905 74 | 0.412 0.0074 41 | -0.220 0.1670 41 |
| Gender | Correlation Coefficient Significance Level P n | -0.232 0.0425 77 |  | -0.020 0.8645 77 | -0.157 0.2076 66 | 0.010 0.9306 76 | 0.315 0.0063 74 | 0.387 0.0006 74 | 0.143 0.2247 74 | 0.363 0.0015 74 | -0.003 0.9824 74 | 0.069 0.6680 41 | 0.046 0.7751 41 |
| eGFR | Correlation Coefficient Significance Level P n | 0.183 0.1114 77 | -0.020 0.8645 77 |  | -0.357 0.0033 66 | 0.097 0.4040 76 | 0.047 0.6933 74 | -0.093 0.4323 74 | 0.188 0.1090 74 | -0.067 0.5678 74 | -0.717 <0.0001 74 | 0.583 0.0001 41 | -0.191 0.2322 41 |
| Urinary protein | Correlation Coefficient Significance Level P n | -0.145 0.2466 66 | -0.157 0.2076 66 | -0.357 0.0033 66 |  | 0.004 0.9752 65 | -0.042 0.7411 63 | 0.124 0.3339 63 | -0.194 0.1276 63 | 0.227 0.0730 63 | 0.285 0.0234 63 | -0.210 0.1885 41 | 0.312 0.0471 41 |
| Transplant era | Correlation Coefficient Significance Level P n | -0.129 0.2672 76 | 0.010 0.9306 76 | 0.097 0.4040 76 | 0.004 0.9752 65 |  | -0.172 0.1445 73 | -0.147 0.2158 73 | -0.364 0.0015 73 | -0.065 0.5835 73 | -0.106 0.3709 73 | 0.290 0.0699 40 | -0.235 0.1442 40 |
| C3M | Correlation Coefficient Significance Level P n | -0.128 0.2776 74 | 0.315 0.0063 74 | 0.047 0.6933 74 | -0.042 0.7411 63 | -0.172 0.1445 73 |  | 0.654 <0.0001 74 | 0.381 0.0008 74 | 0.634 <0.0001 74 | 0.043 0.6809 94 | 0.282 0.0317 58 | -0.164 0.2303 55 |
| C4M2 | Correlation Coefficient Significance Level P n | -0.101 0.3902 74 | 0.387 0.0006 74 | -0.093 0.4323 74 | 0.124 0.3339 63 | -0.147 0.2158 73 | 0.654 <0.0001 74 |  | 0.174 0.1380 74 | 0.801 <0.0001 74 | 0.097 0.4116 74 | 0.062 0.7109 38 | 0.045 0.7876 38 |
| C5M | Correlation Coefficient Significance Level P n | 0.106 0.3684 74 | 0.143 0.2247 74 | 0.188 0.1090 74 | -0.194 0.1276 63 | -0.364 0.0015 73 | 0.381 0.0008 74 | 0.174 0.1380 74 |  | 0.189 0.1066 74 | 0.003 0.9773 74 | -0.088 0.5978 38 | 0.084 0.6182 38 |
| C6M | Correlation Coefficient Significance Level P n | -0.140 0.2355 74 | 0.363 0.0015 74 | -0.067 0.5678 74 | 0.227 0.0730 63 | -0.065 0.5835 73 | 0.634 <0.0001 74 | 0.801 <0.0001 74 | 0.189 0.1066 74 |  | 0.245 0.0355 74 | 0.112 0.5017 38 | 0.113 0.4976 38 |
| Pro-C6 | Correlation Coefficient Significance Level P n | -0.125 0.2905 74 | -0.003 0.9824 74 | -0.717 <0.0001 74 | 0.285 0.0234 63 | -0.106 0.3709 73 | 0.043 0.6809 94 | 0.097 0.4116 74 | 0.003 0.9773 74 | 0.245 0.0355 74 |  | -0.374 0.0010 74 | 0.182 0.1829 55 |
| uC3M | Correlation Coefficient Significance Level P n | 0.412 0.0074 41 | 0.069 0.6680 41 | 0.583 0.0001 41 | -0.210 0.1885 41 | 0.290 0.0699 40 | 0.282 0.0317 58 | 0.062 0.7109 38 | -0.088 0.5978 38 | 0.112 0.5017 38 | -0.374 0.0010 74 |  | -0.156 0.1916 72 |
| uPro-C6 | Correlation Coefficient Significance Level P n | -0.220 0.1670 41 | 0.046 0.7751 41 | -0.191 0.2322 41 | 0.312 0.0471 41 | -0.235 0.1442 40 | -0.164 0.2303 55 | 0.045 0.7876 38 | 0.084 0.6182 38 | 0.113 0.4976 38 | 0.182 0.1829 55 | -0.156 0.1916 72 |  |
