# Supplementary figures and images for: Non-invasive quantification of collagen turnover in renal transplant recipients
Source: PLoS One. 2017 Apr 21;12(4):e0175898. doi: 10.1371/journal.pone.0175898 (PMC5400243; doi:10.1371/journal.pone.0175898)

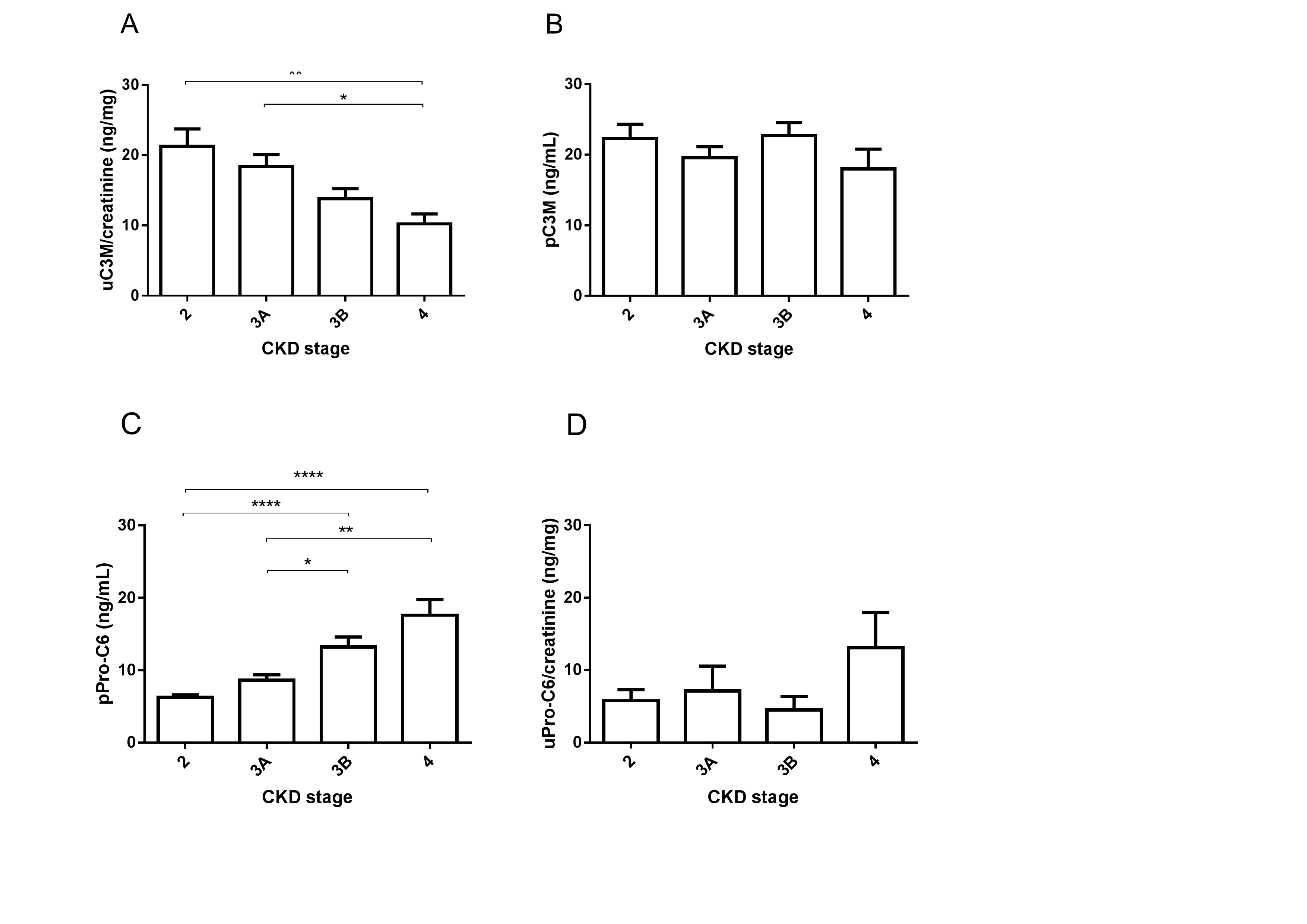

Supplement: S1 Fig — Levels of uC3M/creatinine (A), pC3M (B), uPro-C6/creatinine (C) and pPro-C6 (D) in RTR divided into CKD stage 2 to 4. Statistical differences were assessed by Kruskal Wallis test and Dunn's multiple comparison post-hoc test. Asterisks indicate statistical significance between specified groups as indicated by bars. (* = p<0.05; ** = p<0.01; **** = P<0.0001). (TIF) [file pone.0175898.s002.tif]
